# Grief, disorientation, and futurity

**DOI:** 10.1007/s11097-021-09752-z

**Published:** 2021-06-18

**Authors:** Constantin Mehmel

**Affiliations:** School of Philosophy & Art History, University of Essex, Wivenhoe Park, Colchester, CO4 3SQ UK

**Keywords:** Grief, Disorientation, Self-understanding, Foreclosed future, Futural self, Heidegger

## Abstract

This paper seeks to develop a phenomenological account of the disorientation of grief, specifically the relationship between disorientation and the breakdown in practical self-understanding at the heart of grief. I argue that this breakdown cannot be sufficiently understood as a breakdown of formerly shared practices and habitual patterns of navigating lived-in space that leaves the bereaved individual at a loss as to how to go on. Examining the experience of losing a loved person and a loved person-to-be, I instead propose that this breakdown should be understood primarily in relation to a distinctive kind of futurity operative in disorientation, *irrespective* of the extent to which there is a breakdown of formerly shared practices and habitual patterns of navigating lived-in space. Drawing on the resources afforded by Heidegger’s phenomenology, I argue that it is a *core characteristic* of the experience of disorientation in grief that the grieving person can no longer meaningfully press ahead into a *specific futural self*. This view comes with certain advantages over existing accounts of the temporality of grief for making sense of the disorientated relationship to futurity, which the appeal to Heideggerian resources makes possible.

“It is clear that, though it is not always, the loss of loved ones can be deeply disorientating,” philosopher Harbin writes, “from the disruption of everyday habits one has developed in relation with the person, to the sudden need to reconsider one’s most substantial life plans.” (2016: 53) That the loss of a loved one can prompt a breakdown in the bereaved’s practical self-understanding, to the effect that the bereaved is left at a loss as to how to go on, seems clear. Yet surprisingly, grief’s disorientating effects – specifically, the relation between disorientation and the breakdown in practical self-understanding that is central to grief – have not received much explicit phenomenological attention. In addition, the study of grief is typically concerned with the case of losing a partner, a friend, or a child, with a focus on formerly shared practices and habitual patterns of navigating lived-in space now disrupted, as illustrated by Harbin’s remarks. However, there are also other possible sources of the disorientation of grief than the loss of a significant other with whom one had shared a significant life span, like the experience of stillbirth, that significantly complicate such a focus. In this paper, I seek to develop a phenomenological account of the disorientation of grief that is not limited to the case of losing a loved person. I propose that a comparative phenomenological analysis of both, the loss of a loved person *and* the loss of a loved person-to-be, helps elucidate how best to conceptualise the relationship between disorientation and the breakdown in practical self-understanding at the heart of grief; a conception which has explanatory potential with respect to different sources of the disorientation of grief. Broadening the scope of analysis has important implications for the study of the phenomenology of disorientation of grief in particular and the phenomenology of grief in general.

I proceed as follows. In Sect. 1, I bring more closely into focus the phenomenon and consider two vignettes that depict the experience of losing a loved person and a loved person-to-be respectively. At the heart of the bereaved’s disorientation is a breakdown in the previously taken-for-granted self-understanding that leaves them at a loss as to how to go on. In Sect. 2 and 3, I critically engage with and further develop two possible approaches to understand this breakdown as gestured towards in the recent work of Michael Cholbi. In Sect. 2, I demonstrate that the *difference* between the two vignettes shows that this breakdown cannot be sufficiently understood as a breakdown of formerly shared practices and habitual patterns of navigating lived-in space. In Sect. 3, I argue that the *common* aspects indicate that this breakdown should better be understood primarily in relation to a distinctive kind of futurity in disorientation, *irrespective* of the extent to which there is a breakdown of formerly shared practices and habitual patterns of navigating lived-in space. In Sect. 4, I draw on the resources afforded by Heidegger’s phenomenology and argue that it is a *core characteristic* of the disorientation of grief in both cases that the bereaved can no longer meaningfully press ahead into a *specific futural self*. This view comes with certain advantages over existing accounts of the temporality of grief for making sense of the disorientated relationship to futurity in both cases, which the appeal to Heideggerian resources makes possible.

## The phenomenon in question

The aim of this section is to bring into view the type of experiences that I treat and analyse under the header of “disorientation of grief.” This will prepare the next two sections, in which I explore how best to conceptualise the relationship between disorientation and the breakdown in practical self-understanding at the heart of grief. My aim is not to provide an exhaustive account of the disorientation of grief, let alone of grief *simpliciter*, but rather to identify a *core characteristic* of disorientation that is often involved in grief. To this end, consider the following two vignettes^1^:

1. *The paradigmatic case:* George and Jim have been lovers for more than 10 years now. Jim is George’s first ever male partner and has been of immense support in coming to terms with the realisation that he might also like men; George would not have been able to grow to be as confident a person and as secure in his identity if it had not been for Jim’s continuous love, affection, and support. After three weeks of a visiting research stay abroad, George finds himself counting down the days until he can finally return to their home and hold Jim in his arms again. On the day of his return, George receives a phone call from Jim’s mother that Jim has been fatally injured in a car crash. The news of Jim’s passing leaves George devastated. His world is falling apart. Without Jim around, George feels like everything is off balance and makes life seem impossible to go on. Whilst he goes on in his daily routine – teaching his philosophy class, working out at the gym, shopping at their local supermarket, and so on – he feels like a mere spectator to his own life. He no longer feels at-home in his otherwise all too familiar surroundings: their apartment, their diner next door, their favourite theatre. He feels like a stranger in his own life, everything reminds him of the irrevocable absence of Jim. Who is he, now that Jim has been taken away from him? Why did it have to be Jim who was taken?
2. *The non-paradigmatic case*: Elizabeth is in her late 30 s, single, and has long given up on the idea of ever becoming a mother. Then, she meets fellow writer Edward, they fall in love, and soon she finds herself pregnant with a little boy. Pudding is his name, or at least that is what they decide to call him until he pops into the world. Elizabeth could not be any happier. As many mothers-to-be, she is getting excited about buying little baby clothes for Pudding, imagining what it will be like to have him around, making up stories of wonderful times ahead, like singing him to sleep or his first day at school. Up until the ninth month of her pregnancy, everything is going comfortably well. It is a very straightforward, happy pregnancy. One day, however, Elizabeth senses Pudding’s absence: He is no longer moving and bouncing around as he usually does. Several check-ups later, Elizabeth and Edward are informed that Pudding has gone. Elizabeth’s world is falling to pieces. It felt like she knew her baby boy, and now she will never get to know him. What could have been a wonderful summer in France or anywhere for that matter has become her biggest nightmare. How could she, the person carrying him around and responsible for keeping him alive, lose him? Feelings of guilt overcome her. She cannot picture herself ever knowing how to go on without Pudding.

Allow me two preliminary remarks. First, in considering the experience of stillbirth, I do not follow existing phenomenological scholarship that tends to restrict their analysis of grief to the case of losing a loved person (what I call *the paradigmatic case*) as opposed to a loved person-to-be, like baby boy Pudding (what I call *the non-paradigmatic case*). This has crucial implications for the topic of study, stipulating what an adequate account of the disorientation of grief has to account for. Whenever I write ‘loved one,’ I shall henceforth refer to both cases, unless otherwise stated. Second, my analysis of both vignettes will be exclusively concerned with the first-person perspective of George and Elizabeth. My aim in this study is to propose one general way of thinking about the disorientation that follows from the loss of a loved one that could, then, be further elaborated and complicated depending on the specific case study and its context. Both the interpersonal and social dimensions will likely have a significant impact on the grieving process, its course and duration (cf. Ratcliffe & Byrne, forthcoming), which becomes particularly salient in the case of stillbirth. Elizabeth’s experience of grief will likely be affected and shaped by the support that Elizabeth’s partner Edward may or may not provide (cf. Cacciatore et al., 2009; Dyregrov & Dyregrov, 2008), but also by the wider social environment upholding certain images of motherhood and the taboo around the subject (cf. Burden et al., 2016; Cacciatore et al., 2013).

With this in place, let me turn to the two vignettes. George has lost his long-term lover Jim, and Elizabeth (alongside Edward) has lost her yet to be born baby boy Pudding. Their grief is an emotional response to a painful and irrevocable loss. They do not simply ‘miss’ their loved one akin to a person or object gone missing (cf. Kelly, 2016); rather, they are faced with the painful realisation that their loved one is and will be forever gone. As noted by many, grief is not a singular emotion that is temporally confined to a moment in time. It is rather, in Fuchs’ words, “a complex and heterogenous process, which proceeds and manifests itself in manifold ways, and which is subject to considerable cultural variation and modification” (2018: 45). The emotional response to, and thus experience of, losing a loved one is likely to encompass a variety of different emotional experiences, such as love, sadness, isolation, despair, guilt, anger, or disorientation. Notice both vignettes indicate multiple, different emotional experiences as part of the bereaved’s grief. For instance, George does not only miss Jim who he has loved and still loves with all his heart, he is also raging with anger that it was Jim who had to die in the car crash. Elizabeth’s grief features guilt.

What is more, they both have in common the experience of disorientation as prompted by the loss of their loved one.^2^ I submit this sense of disorientation is a radically first-personal experience that was not there prior to the loss of the loved one and involves a profound phenomenological alteration in how the bereaved navigates lived-in space in relation to themselves, others, and the world.^3^ Disorientation is *first-personal* in the sense that the bereaved is the subject who is undergoing this experience, and it is *radical* in being first-personal in the sense that, in not knowing how to go on and feeling directionless, the bereaved experiences themselves as confronted by the experience of losing a loved one.^4^ It is a self-regarding phenomenon that has to do, as both vignettes make plain, with the breakdown in the bereaved’s previously taken-for-granted self-understanding that was bound up with their relationship to their loved one. How are we to understand this breakdown such that we can make sense of the disorientation of grief? In the next section, I shall begin addressing this question by examining one prominent approach to understand this breakdown that underlies much of the phenomenological scholarship on the paradigmatic case of grief.

## Disorientation and habitual navigation

To elucidate the phenomenology of disorientation of grief, my analysis is guided by the two vignettes. I take them as representative of many first-personal experiences of *profound* grief. Thus, I do not wish to make any generalised claims about whether or not disorientation is a constitutive feature of all types of grief that are about the loss of a loved one, or only contingently related to them. I nonetheless suspect that disorientation is likely to always occur in at least those profound cases that I am concerned with here.^5^

The recent work of Cholbi (2017, 2019) provides a useful starting point. Cholbi’s primary concern is with the complex process of grief as a whole, rather than the self-regarding component of *disorientation* that is (often) involved in, and one specific emotional component of, grief. Yet his work starts from the same insight: namely, the loss of a loved one prompts a breakdown in the bereaved’s practical self-understanding that was bound up with the relationship to their loved one. In developing this insight, Cholbi gestures towards two different (albeit related) ways in which one might want to understand this breakdown that leaves the bereaved at a loss as to how to go on: (1) in terms of a breakdown of formerly shared practices and habitual patterns of navigating lived-in space bound up with the loved one, and (2) in terms of a foreclosure of future trajectories for their relationship to their loved one. Over the next two sections, I shall critically assess both approaches in detail with regards to their explanatory force for the phenomenology of disorientation of grief and, in doing so, situate them in the broader context of phenomenological scholarship on grief. Note that Cholbi himself does not clearly distinguish between these two approaches. I have identified and dissociated them as two separate approaches for the sake of analytic purposes and because I will argue that disorientation is better understood primarily with reference to a distinctive kind of futurity operative in disorientation, *irrespective* of the extent to which there is a breakdown of formerly shared practices and habitual patterns of navigating lived-in space. In contrast to much of the existing scholarship on grief, therefore, I will shift the analytic focus away from a disturbance to one’s habitual patterns of being-in-the-world to an alteration to futurity in grief, which the consideration of the non-paradigmatic case makes possible. However, as I will show in Sect. 3, Cholbi’s second approach has to be refined and further developed in order to adequately capture the disorientating force of grief.

On the first approach, the loss of a loved one means that the bereaved can no longer participate in various shared practices and is disrupted in their habitual patterns of navigating lived-in space that were bound up with the relationship to their loved one. Cholbi writes:

For the death of someone standing in an identity-constituting relationship with us necessitates a change in what sorts of goods are available to us and what we might hope or plan for. Grief thus *disorients* us inasmuch as patters of feeling and acting with which we are familiar are no longer available to us. Our lives cannot proceed in precisely the same manner as they did before. (2017: 100; italics added)

To illustrate the point I take Cholbi to make, let me return to the first vignette. Take the example of going to the local supermarket. George can no longer participate in their shared activity of doing their weekly shop together. Without Jim, all that is left is the painful reminder of his irrevocable absence when entering ‘their’ local supermarket. Clearly limited in scope, this example is illustrative of a key feature of the phenomenology of grief. As widely noted, with the loss of a loved person, the bereaved undergoes a disturbance of a world of shared practical meaning insofar as it was bound up with and dependent on the deceased in a range of ways (cf. Fuchs, 2018; Ratcliffe, 2015a, 2019, 2020). The more time George spends with Jim and the longer they live together, the more profound the effects on George’s being-in-the-world. Their relationship generates shared routines, habits, and assumptions, and much of George’s experiences, activities, and thoughts are likely to implicate and depend for their intelligibility on Jim in one way or another. With Jim’s death, however, there comes a breakdown of habitual patterns of navigating lived-in space in relation to himself, others, and the world that were bound up with his relationship to Jim. As a result, ‘patterns of feeling and acting with which George is familiar are no longer available to him.’ This resonates with much of the scholarship on grief, notably, the work of Matthew Ratcliffe. Ratcliffe emphasises how grief “undermines the very context within which it arises,” because the deceased was not only a person with whom one engaged in shared practices but also a “condition of intelligibility for that world” (2020: 660). Patterns of acting and sensemaking that would have previously set out possible ways for how to go on, dependent on the deceased, are rendered obsolete. “There is a profound sense of being lost,” Ratcliffe notes, “it is not that the right path cannot be discovered but that there is no path to follow and nowhere familiar to retreat to.” (660–661). More recently still, together with Eleanor A. Byrne, they write: “Meaningful courses of action are no longer set out; what once guided and structured one’s activities and associated emotional responses to situations is absent.” (forthcoming).

Overall, the first approach rightly draws attention to the bereaved’s habitual patterns of navigating lived-in space and the extent to which they break down. As just described, this is because the deceased was both the object of but also condition for the bereaved’s habitual patterns of navigating lived-in space, without which they find themselves at a loss as to how to go on. In what follows, however, I shall argue that this alone is not sufficient for explaining the disorientation of grief. Whereas Ratcliffe and Byrne wager that their analysis “applies in the case of a strong relationship with a long-term partner,” consonant with the literature’s focus on the paradigmatic case, “and that it can apply equally to other kinds of relationships” (forthcoming), I shall demonstrate that the first approach, though applicable, does not have the explanatory force required for the non-paradigmatic case. That is also to say, if we aim to provide an adequate account of the disorientation of grief, specifically, the relationship between disorientation and the breakdown in practical self-understanding involved in both cases, we need to go beyond the first approach. This will become particularly clear when we attend to the *difference* between the two vignettes, which will be the task of the remainder of this section.

Let me begin by returning to the second vignette. I think it is fairly uncontroversial to claim that Elizabeth’s relationship with her baby boy Pudding had generated fewer shared routines, habits, and assumptions (to the extent that we can meaningfully speak of a *shared* nature here) than George’s relationship with Jim.^6^ In addition, less of Elizabeth’s patterns of activities and thoughts were dependent on Pudding for their intelligibility than in George’s case. This is not just because George and Jim were together for more than 10 years and therefore spent more time together. It is also because Pudding was yet to pop into the world such that he could share any routines, habits, or assumptions, as well as affect the possibilities of Elizabeth’s experiential world more widely by way of engaging with her (that is, outside the womb). What this suggests, I submit, is that the disorientation of grief in the non-paradigmatic case cannot be sufficiently accounted for if we primarily focus on formerly shared practices and habitual patterns of navigating lived-in space. For the scope of those that were bound up with and dependent on the deceased is significantly smaller than in the paradigmatic case; and yet, like George, for Elizabeth life seems impossible to go on. This is, of course, not to say that the death of Pudding does not prompt a disruption of certain habitual patterns of Elizabeth. Just think of all the anticipatory habits involved in being pregnant, such as thinking about the mutual future on a daily basis and making up stories of wonderful times ahead, which run empty with the death of Pudding.^7^ However, the point here is not so much whether or not disorientation has anything to do with a breakdown of formerly shared practices and habitual patterns of navigating lived-in space that were partly constitutive of the bereaved’s self-understanding. This is certainly the case.^8^ My point is rather that we need to look *beyond* this feature and thus the first approach if we are to give an adequate account of the disorientation of grief, specifically, the relationship between disorientation and the breakdown in practical self-understanding involved in both cases. Let me further motivate this claim by turning to one of the most striking passages of McCracken’s memoir. There, she reflects on the unique character of her first-personal experience of stillbirth:

After most deaths, I imagine, the awfulness lies in how everything’s changed: you no longer recognize the form of your days. There’s a hole. It’s person-shaped and it follows you everywhere, to bed, to the dinner table, in the car.

For us what was killing was how nothing had changed. We’d been waiting to be transformed, and now here we are, back in our old life. (2009: 97)

In the first half of the passage, McCracken provides a phenomenologically astute assessment of the experience of losing a loved person (‘it’s person-shaped’). It corresponds to our analysis of the first vignette and how much of the bereaved’s experiences, activities, and thoughts implicate and depend on the deceased in one way or another. In fact, this passage points to what many first-personal accounts of the paradigmatic case make plain: namely, the bereaved experiences a profound transformation of their being-in-the-world as a whole (e.g., Lewis, 1966: 11). In the second half, McCracken notes how (somewhat unexpectedly) different her own experience of having lost Pudding is. This is, to be sure, not to say Elizabeth will not undergo a profound alteration of how the world as a whole shows up. However, as her remark of being ‘back in our old life’ indicates, this has less to do with a breakdown of formerly shared practices and habitual patterns of navigating lived-in space. For, as we have seen, the scope of those, bound up with and dependent on Pudding and now disrupted, is much smaller.

## Disorientated relationship to futurity

The previous section has shown that the first approach alone is not sufficient for explaining the disorientation of grief, once the scope of analysis is broadened. Let me therefore turn to the second approach and shift the focus of analysis away from a breakdown of formerly shared practices and habitual patterns of navigating lived-in space. Recall that, on the second approach, the breakdown in practical self-understanding is understood primarily in terms of a foreclosure of future trajectories for the bereaved’s individual’s relationship to their loved one. In his more recent work, Cholbi highlights:

others' deaths foreclose many of the possible trajectories for our relationships with them. The deceased therefore bequeath to us a predicament, namely how to continue a relationship that cannot proceed on precisely the same terms as before. But for beings whose self-understandings are predicated to a large degree on our relationships with others, the deaths of others in whom we have invested our well-being compel re-examination of the trajectories of those relationships. As authors of our autobiographies, the deaths of these others force authorial choices upon us about how our relationships with them shall continue (given that they cannot continue on their former terms). The process of grief, I propose, is our attempt to resolve this predicament. … Our emotional attention is drawn to the deceased individual because we are, however inchoately, attempting to ascertain how we can and will relate to her now and in the future. (2019: 498)

Cholbi rightly points out how grieving problematises the bereaved’s future relationship to their loved one. For instance, George can no longer project himself into the future *qua* partner of Jim in the same ways as before, now that Jim is gone. As a result, Cholbi goes on to note, grief forces the bereaved to figure out how to press ahead into the future and thus continue their relationship to their loved one. In his words: ‘As authors of our autobiographies, the deaths of these others force authorial choices upon us about how our relationships with them shall continue.’ This is a striking characterisation of the experience of losing a loved one. It presents grief as a process of coming to terms with the new situation the bereaved is thrown into. It might be phenomenologically plausible to characterise the process of grief *as a whole* in such ‘orientating’ terms, resolving “how to relate to the deceased going forward.” (2019: 498). However, such a framing does not sufficiently take into account the disorientating force of grief and thus the disorientated relationship to futurity in grief. For one thing, it remains unclear what exactly the change in the structure of temporal experience in grief consists of. Phenomenologically, it is not self-evident what it means to undergo a foreclosure of future possibilities. More crucially, in focusing on the activity of finding ways to relate to the loved one in the future, it downplays the extent to which the loss of a loved one radically affects how one can press ahead into the future at the time of feeling disorientated. I contest that if we ever were authors of our autobiographies, what is characteristic of disorientation is precisely that it makes the bereaved unable to meaningfully be the author of their autobiography in the sense that they experience their future as *foreclosed*.^9^

In what follows, I shall carefully advance this claim by attending to a distinctive kind of futurity that is operative in the disorientation of grief. In examining this disoriented relationship to futurity, I shall thus refine and further develop the second approach of tying the breakdown in practical self-understanding to a foreclosure of future trajectories for their relationship to their loved one. Let me begin by returning to an illuminating passage in McCracken’s memoir:

we decided as we wept we would go somewhere we’d never been as soon as we could … Barcelona, maybe. We pictured ourselves walking beneath a hot, unfamiliar sun, somewhere where drinks where plentiful and not made in France. We believed that a short while devoted to oblivion and beauty would make us feel better. We thought that we *could* feel better. Soon enough the notion seemed ludicrous, and we forgot about our Spanish plans. (2009: 10)

This passage suggests that her breakdown in self-understanding, affecting the way she navigates lived-in space and thus the world as a whole shows up to her, involves a distinctive relationship to futurity. Specifically, it conveys a sense in which McCracken experiences herself as closed off from a future in which things could be otherwise, to the effect of not knowing how to go in the present. The disorientation of grief does not only comprise a breakdown in habitual patterns of navigating lived-in space, affecting which possibilities are still open to the bereaved, but crucially also a sense of foreclosed future, affecting how those remaining possibilities are experienced. How, then, are we to understand this *sense of foreclosed future* that I take to be a *core characteristic* of the disorientation of grief? Though this temporal dimension is expressed in many first-personal accounts of grief that involve what I call the experience of disorientation, there has been little phenomenological research that explicitly concerns the experience of futurity in grief; with a few exceptions including Ingerslev (2018), Ratcliffe (2012, 2015b), and Fuchs (2018). Engaging with their work, I will cultivate the interpretive difficulty of capturing this sense of foreclosed future in grief. Specifically, I will rule out two possible hypotheses found in existing phenomenological descriptions before I, then, clear the way for an alternative view.

First, I turn to Fuchs’ account (2018). Fuchs stresses that the temporality of grief is marked by the experience of desynchronisation: “a separation of two forms of time, one flowing, one arrested, which become more and more desynchronized” (50). Whereas other people continue to inhabit a shared temporal world, the bereaved finds themselves painfully stuck in the present. Fuchs demonstrates how the bereaved’s sense of time was dependent on their relationship with their loved person, to the effect that their death yields a sense of being out of time. He cites Denise Riley’s painful report of losing her adult son that leaves her “being cut off from any temporal flow” (2012: 7). Of interest to the present study, Fuchs goes on to note this experience also affects the bereaved’s experience of futurity: “the future is no longer experienced as an open horizon of possibilities and projects” and “bereaved persons abstain from making plans or projects and from taking part in the ongoing life of others” (2018: 51). Fuchs does not say much more about this alteration to the experience of futurity, but his remarks gesture towards a characterisation of the future as closed off and blocked, as lacking openness.

This view captures the painful texture of the bereaved’s experience of the future marked by the absence of their loved one and thus a “no longer.” However, I do not think that such a view, without further specification, is phenomenologically quite right. Attending to the above McCracken passage, it seems clear that, though she does not know how to go on in the present, Elizabeth can still make plans about what she would like to do in the future. She can continue to engage in most future projects, barring those projects which involved preparing for Pudding’s arrival. The disorientated relationship to futurity operative in our case study does not amount to an inability to make plans for the future in light of which the bereaved could attempt to navigate their situation. The bereaved individual might abstain from making any plans; however, they are still able to make plans. This indicates they still experience their future as holding possibilities open to them, contrary to Fuchs’ remark of the future being ‘no longer experienced as an open horizon of possibilities and projects.’ The disorientation is far-reaching and experienced in general, however it does not completely undermine the bereaved’s agency. For instance, although concurrent to his disorientation, George is still able to navigate his role as a philosophy teacher and as such feels orientated in relation to this specific situation. On this interpretation, therefore, the sense of foreclosed future operative in the disorientation of grief does not amount to losing all futural possibilities. The bereaved has not lost access to all futural possibilities *simpliciter*.

An alternative characterisation of the sense of foreclosed future might be as follows: Although the bereaved can still make future plans, none of these futural possibilities show up to them as meaningful any longer. They experience a closing down of *meaningful* futural possibilities. This view can be found in Line R. Ingerslev’s account of the temporality of grief who, like Fuchs, draws on Riley’s profound account of temporal experience in the aftermath of losing her son. Ingerslev notes, “without you, time cannot move and I cannot inhabit this world as a meaningful one” (2018: 351) and “when we lose someone, nothing can happen anymore since we are stuck in time and incapable of expressing and finding meaning … not allowing me to enter into a meaningful future” (353). However, such a phenomenological characterisation cannot fully explain the disorientated relationship to futurity in grief either. In our case study, the bereaved can still distinguish between better or worse things in the future. In her memoir, McCracken stresses how meeting other people in the weeks after Pudding’s death was “excruciating” (2009: 39), something that she tried to avoid as much as possible. In other words, she *is* choosing a future in which she does not have to endure so many painful encounters with other people that remind her of the loss of Pudding in one way or another. The experience of her future as foreclosed does, therefore, not amount to a closing down of meaningful futural possibilities *simpliciter* either.^10^

The point is, rather, that those meaningful futural possibilities still open to the bereaved do not have an impact on their current being-in-the-world robust enough to transform their painful situation and make them feel less, or even no longer, disorientated. This is, of course, not to say that we cannot imagine certain plans that, once actualised at a certain point in the future, might bring *momentary*, *passing* joy to the bereaved’s life. However, this is not the same as the bereaved becoming orientated anew, insofar as their experience of disorientation has to do with the loved one being forever gone. In such cases, Matthew Ratcliffe notes, “there is an intact sense that things could be practically significant – the problem is that they are not” (2012: 125). The bereaved does not lose the capacity to find things practically significant, but things no longer show up to the bereaved in the same ways as before. The meaningful futural possibilities still open to the bereaved no longer allow for the bereaved’s self-realisation of taking themselves to be a certain person in any positive way, to the effect that they feel disorientated going forward. I submit this is how the disorientated relationship to futurity should best be interpreted; already note that I will unpack this hypothesis in the next section with appeal to Heideggerian resources. Ratcliffe shows how one’s experience of futurity is shaped by “a sense of the ongoing projects and commitments that render things significant to us” and, importantly, “constitute a sense of working towards something, a teleological direction” (122). In those cases of grief in which the bereaved still has the capacity to find things practically significant, he goes on to note, the bereaved “retains a sense of teleological time,” and yet, “her long-term future, the ‘personal future’ … appears directionless, difficult, daunting and uncertain” (126). This provides a key insight, namely, that the disorientated relationship to futurity centrally concerns one’s personal future that is foreclosed. But how exactly are we to understand this *personal future*? And how are we to understand that the breakdown in practical self-understanding – one such project that, with Ratcliffe, contributes to one’s longer-term teleological direction and now disrupted – makes one’s personal future appear foreclosed? I propose that Martin Heidegger, one of the phenomenologists influencing Ratcliffe’s own view, can help us here. Drawing on Heideggerian resources, in the next section I will provide an answer to these questions by way of providing an account of how exactly futurity relates to one’s self-understanding and the extent to which one feels orientated in navigating lived-in space.

However, let me conclude this section by addressing one important concern regarding the mode of generality of the preceding interpretation of the disorientated relationship to futurity in grief. One might, and rightly so, note there will be cases of profound grief in which the bereaved *does* lose the capacity to find things practically significant. Ratcliffe attributes Riley’s experience of foreclosed future (that underlies both Fuchs’ and Ingerslev’s analysis) to such a loss, affecting both “long-term experience because there is no longer a teleological sense of direction, of moving or being able to move ‘forward’,” and “short-term experience, as the transition from moment to moment does not include a sense of meaningful change” (2015b: 199). Two things can be said in response: First, one might want to question the claim that Riley’s experience does not include a sense of meaningful change. Remarks such as “because to concede at the outside that it’s ‘indescribable’ would only isolate you further, when coming so close to your child’s death is already quite solitary enough” (2012: 16) indicate that Riley hopes for a future in which she feels capable of writing about the death of her adult son and thus less isolated.^11^ This at least complicates Ratcliffe’s analysis of those cases in which the bereaved loses the capacity to find things practically significant, inasmuch as it might not fully account for the sense of foreclosed future that Riley is speaking to. Second and more important, however, it is proof of the heterogeneity of temporal experience in grief, the acknowledgement of which further complicates Fuchs’ and Ingerslev’s views on futurity in grief. This is because temporal experience can take different forms in profound grief, *including* those illustrated by the McCracken case study however not accounted for by either Fuchs or Ingerslev. I shall restrict my analyses to those cases in which the bereaved is still capable of finding things practically significant, as is the case in our two vignettes.

## Futural self-understanding

Over the last two sections, I have identified what an adequate conception of the relationship between disorientation and the breakdown in practical self-understanding needs to achieve, especially if what we are interested in is to develop a phenomenology of disorientation of grief that is not limited to the paradigmatic case. The *difference* between the two vignettes has indicated that the breakdown in self-understanding cannot be sufficiently understood in terms of a breakdown of formerly shared practices and habitual patterns of navigating lived-in space. It should rather be primarily understood in relation to futurity, as the *common* aspects between the two vignettes have indicated. Close analysis of both vignettes has shown that we are in need of a view of the disorientation of grief that links the breakdown in self-understanding to the distinctive kind of futurity operative in disorientation, *irrespective* of the extent to which there is a breakdown of formerly shared practices and habitual patterns of navigating lived-in space.

To achieve this task, let me now turn to Heidegger’s account of futural self-understanding in *Being and Time* (1996). My aim here is not to give an exhaustive interpretation but, rather, to draw on some Heideggerian resources with the aim of providing an adequate conception of the relationship between disorientation and the breakdown in self-understanding – a conception that, as I have shown, needs to integrate the sense of foreclosed future as a *core characteristic* of the disorientation of grief.^12^

For Heidegger, what it means to understand ourselves is not (primarily) a matter of reflective, deliberative self-conception. It is, rather, a matter of making sense of ourselves by embodying a certain way of life. As *Dasein* (i.e., the kind of being we are that understands itself as being at issue in everything it does), we pre-reflectively understand ourselves through the ability to skillfully “press ahead” into a certain way of life, which discloses the world as an arena for meaningful action within which we can exercise our existence and make sense of ourselves accordingly. To press ahead into a way of life, then, is to have and exercise the relevant abilities and skills to be this or that (1996: 134–145). Note that we can only press ahead into those possible ways of life that already show up to us as affectively mattering. Take our first vignette. George would not project himself onto the possibility of being a partner if this possibility had not already shown up to him as mattering. He does so, however, because being Jim’s partner matters to him and thus drawing him to act in certain ways. In light of his love for Jim, George pre-reflectively understands himself as being a partner.^13^ His life is organised around his *orientating* concern for what it means to be a partner, which guides him in how he navigates lived-in space in relation to himself, others, and the world. We can helpfully think of this background orientation, as B. Scot Rousse (following Charles Taylor) suggests, akin to how we navigate a city:

The way we are guided by such a tacit and individualized normative orientation is analogous to the way in which we talk about having a ‘sense of direction’ in a city, even though we might not be able to draw a detailed representation or map. (2019: 172)

On the here-proposed view, then, our pre-reflective self-understanding gives us direction in life. Note that this should not be understood in static, fixed terms, however. Recall that Jim was George’s first male partner and that it took George quite some time to come to terms with the realisation that he might also like men. George’s ability to be a partner, and thus his self-understanding as being a partner, has transformed throughout the time of their relationship. The more we press ahead into a possible way of life and exercise a specific for-the-sake-of-which, according to Heidegger, the more competent do we become in navigating lived-in space as being such (1996: 139). We gain a better sense of direction and thus feel more orientated in embodying a certain way of life. Hence, Mark Wrathall notes:

Commitments to a particular course of action contribute to the function of world disclosure by involving us in the significations of the world in such a way that we own or appropriate them, develop them, and, in the process of developing them, enrich our skills for coping by giving us a more precise ability to anticipate and respond to the solicitations of our actual situation. (2013: 196)

Importantly, attending to the temporally extended character of pressing ahead into a specific self-understanding is only one part of Heidegger’s account. It appears to prioritise past experiences and the acquisition of certain habitual patterns of navigating lived-in space across time for our self-understanding, so that one may wonder how a Heideggerian conception of self-understanding could help in advancing a phenomenology of disorientation of grief that is not limited to the paradigmatic case. However, Heidegger is clear that our self-understanding is *futural*; in fact, he is arguing for the primacy of the future in our self-understanding (1996: 301). Now, we need to be careful in how to understand this future-directedness. It is not to be understood in terms of us projecting ourselves in the future as if on a chronological timeline in what Heidegger calls ‘ordinary temporality.’ What it means for George to be a partner is not an attainable goal he could ever exhaustively reach. Instead, both the past and the present show up to him in light of his ongoing, open-ended concern for what it means to be a partner. For instance, George is thinking of buying a present for his partner Jim precisely for-the-sake of being a partner; this plan shows up as meaningful to him because he presses ahead into being a partner. Although dependent on the past into which we are thrown and the shared present we experience with others, our pre-reflective self-understanding is ultimately determined by the for-the-sake-of-which we press ahead into. This is what Heidegger captures with the term ‘originary future,’ “the coming in which Dasein comes towards itself in its ownmost ability-to-be” (1996: 299; translation modified). In pressing ahead into a certain way of life and pre-reflectively understanding ourselves thus, a *specific futural self* is coming towards us, opening up a range of meaningful possibilities in light of our concern for being this or that.

Now that we have advanced a Heideggerian sketch of futural self-understanding, I want to return to the disorientation of grief. Specifically, I want to address the relation between the breakdown in self-understanding and the sense of foreclosed future operative in the disorientation of grief. Once more, let me turn to the two vignettes but this time draw on our Heideggerian resources. As should be clear by now, what it means for both George and Elizabeth to pre-reflectively understand themselves as being a partner and becoming a mother respectively is to press ahead into such a way of life. Their lives are organised around their orientating concern for what it means to be such, which in turn provides them with a sense of direction in their lives. Crucially, their self-understanding is *futural*. Though they can both only press ahead into this way of life because it already affectively matters to them, their current and past experiences show up in light of their concern for whom they take themselves to be, opening up a range of meaningful possibilities.^14^ With the loss of their loved one, however, their pre-reflective self-understanding breaks down; instead, they are confronted with the existential question of how to go on now that their loved one is forever gone. On the Heideggerian picture, we are now in the position to explain their disorientation as follows: The breakdown in self-understanding means that the bereaved can no longer meaningfully exercise a specific for-the-sake-of-which, a specific self-understanding. George and Elizabeth can no longer meaningfully press ahead into being a partner and becoming a mother respectively. What exactly does this mean? To begin, it does not mean that their self-understanding as being a partner and a mother completely collapses in the sense that they would no longer be guided by their love for their loved one. On the contrary, their loved one continues to be the basis on which they seek orientation in life; in fact, we might reasonably say that the bereaved only now comes to fully realise the deceased’s significance in and for their life.

The point is rather that, although they are still committed to this specific self-understanding, this specific self-understanding is no longer possible. This is what it means that they are no longer able to meaningfully press ahead into this way of life. As a result, the bereaved no longer has this *specific futural self* coming towards them that could guide them in navigating lived-in space in relation to themselves, others, and the world in such ways that would allow for the bereaved individual’s self-realisation. The coming towards of this specific futural self, however, is the condition for the bereaved to experience their situation as holding meaningful possibilities for their self-realisation.^15^

Ultimately, appealing to Heideggerian resources makes possible a phenomenological description of the bereaved individual’s experience of their future as foreclosed in terms of a loss of a *specific futural self*, which comes from a breakdown in their previously taken-for-granted self-understanding. I submit that it is in this sense that we can make sense of Ratcliffe’s claim that in those cases of grief I am concerned with here, the bereaved retains a sense of teleological time, all the while experiencing their personal future as directionless. This is because without this specific futural self coming towards the bereaved, they can no longer meaningfully incorporate the possibilities still open to them into their self-understanding they are still committed to that, however, is no longer possible. Hence their personal future appears directionless; they feel disorientated in navigating lived-in space, including the feeling as though they are no longer present in their activities.

This view, then, comes with certain advantages over existing accounts of the temporality of grief for making sense of the disorientated relationship to futurity in both cases. Disorientation does not mean that the bereaved cannot make any plans or engage in any projects (cf. Fuchs), nor that they experience their future as without any meaningful possibilities open towards them (cf. Ingerslev). Rather, it means that a specific future drops out which significantly impacts one’s personal future. To reiterate: From the fact that George and Elizabeth are unable to continuously meaningfully press ahead into being a partner and becoming a mother respectively, it does not follow that they are unable to press ahead into anything at all. As Heidegger makes clear, pressing ahead into the future is a *constitutive* feature of what it means to be the kind of being we are (1996: 135) This corresponds to my critical engagement with existing phenomenological accounts of temporal experience in grief, precisely because the future does not collapse entirely. Insofar as their overall navigation of lived-in space is dominated by their desire to have their loved one back, however, they will nonetheless experience their future – specifically, their personal future – as foreclosed. As Kelly puts it, "[the] griever, seeing the world as a world in which his deep love finds its object no longer, puts the past be*fore*, in front of, himself” (2016: 176).

To conclude: In framing the breakdown in self-understanding at the heart of disorientation primarily in relation to the distinctive kind of futurity involved, specifically focusing on the primacy of the future in our self-understanding, this Heideggerian proposal can account for both the paradigmatic and non-paradigmatic case of grief. It is compatible with the focus on a breakdown of formerly shared practices and habitual patterns of navigating lived-in space; and yet, crucially, it goes beyond this by integrating the *sense of foreclosed future* as a *core characteristic* of the disorientation of grief, *irrespective* of the extent to which there is such a breakdown. Let me end this paper by raising two possible concerns one might have: one regarding the core characteristic of foreclosed future and another regarding the conceptual focus on self-understanding and futural self.

First, the here-proposed conception hinges on the claim that the experience of foreclosed future is a core characteristic of the disorientation of grief. Yet, what if the future is not always experienced as radically foreclosed as required by my conception? Suppose a friend of George has lost his father. He is thrown off balance for years but, eventually, starts gaining a new orientation. Given his own emotional journey, he kind-heartedly tells George (however insensitive we might think such ‘advice’ is) that he will eventually feel less disorientated. George is told he could find a way of continuing his bond with Jim that would ‘heal’ his breakdown in self-understanding in a way that acknowledges Jim’s death without letting go of Jim and his importance for his life. If this were the case, however, one might object that the future was not as radically foreclosed in and during George’s experience of disorientation. Two things can be said here. For one, as soon as new meaningful futural possibilities show up to him that would allow for his self-realisation, I submit, his experience can no longer be characterised as one of disorientation in the here relevant sense. This is because by then he would have managed to begin cultivating a new self-understanding that would, in turn, provide new guidance for navigating lived-in space. What this suggests, for another, is that the possibility of cultivating a new self-understanding is not available to him at the time his friend gives the advice, precisely because he experiences his future as foreclosed.^16^

Second, in foregrounding self-understanding, my conception has focused on the self-regarding aspects of grief, specifically emphasised the loss of a specific futural self, irrespective of the extent to which there is a breakdown of shared practices and habitual patterns of navigating lived-in space bound up with and dependent on the deceased in a range of ways. One may thus worry that the relational aspects of one’s self-understanding, which distinguish the disorientation of grief from other kinds like the disorientation following from one’s job, become secondary to self-understanding, making the here-proposed conception too self-centered. Let me therefore end by emphasising that (interpersonal) grief concerns the loss of a loved one – one who was not only a medium for but *at the very heart* of one’s self-understanding, making possible the coming towards of a specific futural self and shaping it over time. While my aim in this paper has not been to provide an exhaustive account of the disorientation of grief, elaborating on these relational aspects of self-understanding would strengthen the Heideggerian conception that I have developed here.^17^

## Data Availability

Not applicable

## Footnotes

1 The first vignette is inspired by Isherwood’s novel (2010) *A Single Man*, and slightly adapted for present purposes. In contrast, the second vignette is based on McCracken’s heart-wrenching memoir (2009) *An Exact Replica of a Figment of My Imagination*. To be clear, it is not for me to determine whether or not Elizabeth McCracken would describe her own experience as one of *disorientation*; that is, I do not want to, let alone am I in the position to make any prescriptive claims as to how she did feel or should have felt. Instead, my point is that her story displays certain tendencies that might be described and analysed under the header of ‘disorientation of grief.’ To avoid confusion, I will use the name McCracken whenever I am directly engaging with her memoir. All other references will be to my vignette character Elizabeth as based on the memoir.

2 As will become clear throughout this paper, the here-proposed account of disorientation differs from Ahmed’s conception of disorientation (2006) in that I am less concerned with the *spatial* extension of bodies but, rather, with the *temporal* dimension of disorientation, specifically, a *distinctive kind of futurity* that is operative in the disorientation of grief. A detailed engagement with her work is beyond the scope of this paper, however.

3 Broadly, I understand lived-in space – in contrast to physical or geometric space – as the felt, experiential space we inhabit. Compare Fuchs’ definition: “Lived space may be regarded as the totality of the space that a person prereflectively ‘lives’ and experiences, with its situations, conditions, movements, effects and its horizon of possibilities – meaning, the environment and sphere of action of a bodily subject.” (2007: 426).

4 This first-personal nature brings to mind the description of irony by Lear: “In a nutshell, the experience of irony is a peculiar experience that is essentially first-personal: not simply in the sense that all experience is the experience of some I, but that in having an experience of irony I experience myself as confronted by that very experience.” (2011: 9; see also Ingerslev, 2018: 349–354). Ultimately, however, the experience of irony instils us with a sense of direction and is therefore forward-moving whereas the experience of disorientation leaves us paralysed and stuck in the present. This will become clear in Sect. 3.

5 Although I leave aside for now an argument for this view, consider the following scenario. Suppose someone is grieving in response to the death of a person who, though loved, had not been a source of orientation for the bereaved individual. It seems plausible to claim that the bereaved will likely experience what are often identified as other-regarding elements of grief (e.g. wanting to promote the deceased’s interests, feeling sad at their loss), without, however, undergoing the self-regarding experience of disorientation. For a similar argument in relation to security-based attachment, see Wonderly (2016: 236–240).

6 Speaking of ‘fewer’, I do not wish to suggest that those shared practices are *qualitatively* less intensively experienced. Importantly, I do not wish to downplay the unique bodily, affective, and relational dimensions of pregnancy.

7 Based on her interviews with women experiencing stillbirth, Esther Lea Kint notes how such a loss “resulted in a complex bereavement experience whereby the women grieved for multiple losses, including the loss of their baby and the loss of their future self (i.e., motherhood).” (2015: 103). One of her interview excerpts reads as follows: “It wasn’t just the loss of Charlotte, it was the loss of all my hopes and dreams. Wanting to be a mother for so long, having her in me for so long, knowing all her little quirks when she moved … and then suddenly her not being there. She was my first child.” (103) This suggests a close connection between the loss of anticipatory habits and the loss of a future self. In Sect. 4, I will draw on Heideggerian resources to identify what I call the loss of a *specific futural self* as a *core characteristic* of the disorientation of grief.

8 See, for instance, the feminist phenomenological exploration of the lived experience of the pregnant and birthing woman by Verhage (2014).

9 Line R. Ingerslev makes a similar point concerning Peter Goldie’s narrative account: “It seems to be part of Goldie’s narrative approach that grieving is a way of coming to understand this process as one’s own grief and thereby grief becomes a way of coming to terms with one’s new situation. Thereby, grief is the experience of a potential therapeutic or healing process that allows us to grasp it as such, when we are somehow passed it. On the other hand, therefore, following the narrative account might add too much emphasis on exactly the having-to-come-to-an-end of a narrative. … Grief questions my survival in this radical sense and therefore I am brought to a halt, since I cannot respond.” (2018: 349–350) That said, I think Ingerslev’s own analysis of ‘being brought to a halt’ requires some refining, to which I shall come shortly.

10 Along the same lines, Owen et al. (2015: 173–176) note that the experience of ‘not seeing a future’ in depression does neither involve the collapse of the future nor a normatively flat future; corresponding to the two hypotheses that I have ruled out concerning the temporal experience in grief. They explicitly note: “For we have seen evidence that D4 *does* recognise normative differences in his future: He clearly prefers the future in which the present interview ends sooner rather than later and in which he is able to get back to his room.” (176; italics original).

11 Again, this corresponds to Owen et al.’s analysis of temporal experience in depression, cf. footnote 10.

12 My reconstruction is particularly indebted to the reading of *Being and Time* by Blattner (1999).

13 This, of course, does not preclude moments of reflective, deliberative self-conception as, for instance, in those conversations where George and Jim discuss the status of their relationship. However, it is not reducible to this.

14 One might worry (thanks to reviewer 2 for pointing this out) that a Heideggerian conception of self-understanding cannot satisfactorily account for the non-paradigmatic case because pressing ahead into a certain way of life presupposes already existing sets of abilities and skills to be this or that, however becoming a parent is a transformative experience and, in this sense, precisely not a pressing ahead on the basis of pre-existing sets of abilities and skills. Two things can be said in response: First, though pressing ahead is to have and exercise the relevant abilities and skills to be such, it should not be understood as a two-stage process. Rather, it is an ongoing and open-ended concern of developing, working out, and exercising the relevant abilities and skills, the process of which can include the transformative overhaul of previous forms of self-understanding that pregnant women report can come both with the birth of the baby but also with the experience of pregnancy itself. What it does presuppose, however, is that the possibility to be such shows up as affectively mattering to one. For, otherwise, it would not show up as a possible way of life for one to press ahead into. This is why, second, Elizabeth can press ahead into the possibility of becoming a mother because of her love for Pudding. In fact, the reason why Elizabeth feels disorientated is precisely because she had projected herself onto this way of life (and thus the futural possibility of being a mother) – the possibility of which had provided her with guidance in navigating lived-in space – which is now no longer possible. This is especially the case insofar as, on the Heideggerian conception, one’s self-understanding is *futural*; it is about who we take ourselves to be.

15 It should therefore be clear that, although the disorientation of grief as construed with appeal to early Heidegger might *prima facie* remind us of Heideggerian Angst, there are a number of principal differences. Most notably, in contrast to Heideggerian Angst, in the disorientation of grief the loss of this specific futural self comes with a radical reconfiguration of the bereaved individual’s life-world whilst at the same time making them all the more concerned with their life-world, being constantly reminded of the painful and irrevocable absence of their loved one.

16 Lear (2006) pursues this question of cultivating a new self-understanding at length in his study on the cultural collapse of a Native American tribe, the Crow, whose world and self-understanding became no longer intelligible. I think that Lear’s case is both more extreme and less severe: It is more extreme because Lear depicts a cultural world collapse as opposed to a collapse of a particular futural self. But it is less severe in that there is one significant normative commitment bound up with the Crow’s world that remained: the Crow received a *visionary dream* that allowed them to see a possible Crow future (however vague); a commitment to the Crow future thus remained possible. In contrast, in the here-considered cases, the bereaved individual does not have a dream, or any other extraordinary, supernatural commitments, at the time of feeling disorientated; instead, they experience their future as foreclosed.

17 Thanks to reviewer 1 for flagging the relational aspects of self-understanding.
